# Transfer of Cry1F from *Bt* maize to eggs of resistant *Spodoptera frugiperda*

**DOI:** 10.1371/journal.pone.0203791

**Published:** 2018-09-12

**Authors:** Camila S. F. Souza, Luís C. P. Silveira, Débora P. Paula, David A. Andow, Simone M. Mendes

**Affiliations:** 1 Universidade Federal de Lavras, Lavras, Minas Gerais, Brazil; 2 Embrapa Milho e Sorgo, Sete Lagoas, Minas Gerais, Brazil; 3 Embrapa Recursos Genéticos e Biotecnologia, Parque Estação Biológica, Brasília, Federal District, Brazil; 4 University of Minnesota, St. Paul, Minnesota, United States of America; Ecole des Mines d’Ales, FRANCE

## Abstract

The intergenerational transfer of plant defense compounds by aposematic insects is well documented, and since 2006, has been shown for Cry toxins. Cry toxins are proteins naturally produced by the soil bacterium *Bacillus thuringiensis* (*Bt*) and its genes have been expressed in plants to confer insect pest resistance. In this work we tested if non-aposematic larvae of a major maize pest, *Spodoptera frugiperda*, with resistance to Cry1F, could transfer Cry1F from a genetically engineered maize variety to their offspring. Resistant 10-day-old larvae that fed on Cry1F *Bt* maize until pupation were sexed and pair-mated to produce eggs. Using ELISA we found that Cry1F was transferred to offspring (1.47–4.42 ng Cry1F/10 eggs), a toxin concentration about 28–83 times less than that detected in Cry1F *Bt* maize leaves. This occurred when only one or both sexes were exposed, and more was transferred when both parents were exposed, with transitory detection in the first five egg masses. This work is an unprecedented demonstration that a non-aposematic, but resistant, species can transfer Cry1F to their offspring when exposed to *Bt* host plant leaves as immatures.

## Introduction

Some insect species developed the ability to subvert chemical plant defenses by taking up secondary compounds with relative impunity, instead of detoxifying them [1,2], and then using them for various purposes. These include defense against predation [1], recognition of hosts for oviposition or larval feeding [3], precursors for pheromone synthesis [4] or UV protection [1]. This process has been widely studied in aposematic lepidopterans [5], but has also been observed in other aposematic species [6,7]. Once ingested, the compound is absorbed through the gut membrane (a part also might be excreted and/or degraded), transported into the hemolymph, and deposited in particular sites of the body [1,8]. In some species, these compounds are transferred maternally and/or paternally to the offspring as a part of a defense syndrome to protect eggs and hatching larvae [9,5].

While uptake and intergenerational transfer of secondary plant compounds has been known for decades, the first demonstrations that ingested Cry proteins could be taken up by aposematic insects and transferred to the offspring eggs only occurred about a decade ago [10–13]. Cry proteins are one of the entomotoxins produced by the soil bacterium *Bacillus thuringiensis*, and therefore have also been referred to as *Bt* toxins. According to the ‘classical’ model of mode-of-action of these entomotoxins, after ingestion, they are solubilized in the insect midgut and activated by midgut proteases (cleavage of a terminal end), which enables a domain to interact with cadherin-like receptors on the surface of midgut epithelial cells. This leads to pore formation, causing damage to the midgut epithelium and consequential insect death [14]. Because of the specific toxicity, *cry* genes have been used to genetically transform major commercial crops to have herbivore resistance.

As Cry toxin uptake and intergenerational transfer may have significant implications for ecological risk assessment of genetically engineered *Bt* plants and pest resistance evolution, it is important to determine if non-aposematic species, such as a target-pest of a *Bt* crop, can uptake and transfer the toxin to the eggs. In this work we demonstrated the ability of non-aposematic, resistant *Spodoptera frugiperda* (J.E. Smith) [Lepidoptera: Noctuidae] to transfer Cry1F to its offspring eggs after feeding on *Bt* maize as larvae. The larva is one of the most important pests of maize worldwide, especially in the tropics and subtropics, because it severely defoliates the plants. *Bt* maize varieties expressing Cry1F have been widely used to control this pest. As resistant populations of *S*. *frugiperda* have been reported in different countries [15–17], our findings imply that intergenerational transfer of Cry toxin is likely in *Bt* maize fields.

## Materials and methods

### *Bt* and non-*Bt* maize cultivation

The two varieties of maize (*Zea mays*) used in this work were *Bt* maize expressing the TC1507 trait (OECD unique identifier: DAS-Ø15Ø7–1, also known as Herculex^®^ I, Dow AgroSciences, Indiana, USA), and a near-isogenic variety without TC1507 (non-*Bt* maize). The *Bt* maize variety was genetically engineered for insect-resistance and herbicide-tolerance and expressed the Cry1F and PAT proteins. They were planted in 2015 in an experimental field at Embrapa Maize and Sorghum, Sete Lagoas-MG, Brazil (19°28’30”S, 44°15’08” W, 732 m altitude). Each plot had five rows 5 m long, with interrow spacing of 0.5 m, and 5 plants/m. The soil was a silty red-yellow latosol with medium texture. Fertilization at planting was 400 kg/ha of NPK 8-28-16, and top-dressed with 90 kg/ha of N (200 kg/ha of urea) at 20 days after planting. Leaves were harvested at the V8 stage for use in rearing and experiments.

### Resistant and susceptible *Spodoptera frugiperda* to Cry1F

The Cry1F resistant population of *S*. *frugiperda* used in this work was selected by Leite et al. [18]. It was reared in the laboratory of Ecotoxicology and Insect Management (Embrapa Maize and Sorghum) at 25± 2°C, 12:12 h L:D and 60 ±10% RH, according to the methodology used by the same authors [18]. This population can complete development on excised leaves of *Bt* maize, and resistance is autosomal, incompletely recessive with simple monogenic inheritance [18], similar to resistance in this species found throughout Brazil [15,19,20]. Indeed, eight Brazilian resistant colonies that were independently isolated from all areas of the maize growing region of the country (from Bahia, in the northeast, to Paraná, in the south) were found to carry the same resistance allele [15], so the genetic basis of resistance for *S*. *frugiperda* is well characterized for the Brazilian populations. The susceptible population has been maintained in the laboratory since 1995 at Embrapa Maize and Sorghum predating the occurrence of *Bt* maize in Brazil. This population is the same as the SUS population in [15]. Individual neonates from the resistant and susceptible populations were reared individually for 10 days in 50 ml plastic cups (closed with acrylic lid) with daily supply of leaves (at the V8 stage) from the non-*Bt* maize variety.

### Cry1F exposure

Ten day-old resistant and susceptible larvae were given V8 leaves of *Bt* maize or non-*Bt* maize as sole food until pupation. This larval age was used for the bioassays to give the susceptible larvae a higher chance of surviving *Bt* maize exposure. Leaves were renewed every other day and larval survival was recorded daily. Care was taken to ensure that larval diet did not contaminate pupae or adults. Pupae were isolated from the larval diet, and adults were sexed within 24 h after emergence and held in clean cages until designated to one of four treatments: 1) Susceptible moths with both sexes not exposed to *Bt* maize (control-group); 2) Resistant moths with only males exposed to *Bt* maize as larvae; 3) Resistant moths with only females exposed to *Bt* maize as larvae; and 4) Resistant moths with both sexes exposed to *Bt* maize as larvae. Each treatment had 18 replicate mating pairs. Only resistant larvae were capable of producing adults when exposed to Cry1F, so there was no treatment with susceptible larvae exposed to *Bt* maize. Each couple was held separately in a PVC tube cage (30 cm height and 10 cm diameter) lined with sulfite A4 paper as an oviposition substrate. Each cage contained cotton with a 50% sucrose solution (m/v) containing 5% of ascorbic acid (m/v) as food, and all cages were maintained at 25±1°C, 8:16 h L:D, 50±10% RH. Egg masses were collected daily until female death and were weighed before storing at -5°C for Cry1F detection and quantification.

### Detection and quantification of Cry1F

Ten couples per treatment were selected for detection and quantification of Cry1F. Each selected couple had uninterrupted daily oviposition for five days from the first day of oviposition, and generally had large uniform quality egg masses. The egg masses from the first to the fifth day of oviposition were collected and frozen at -5°C. Before analysis, egg masses were thawed, weighed, and individually washed for 10 min with gentle agitation (150 rpm) in phosphate-buffered saline with 0.1% Tween 20 (1X PBST). Microscopic examination (100X magnification) showed that all surface particulates were removed, including adult scales. Egg masses from each couple and each day were macerated separately using a glass pestle or knitting needle and added to 1000 μl 1X PBST. After maceration, samples were homogenized by vortexing 5 s, and then centrifuged at 15,500x*g* for 20 min at 4°C. Supernatants were transferred to new microtubes and used for Cry1F detection and quantification by sandwich ELISA using Agdia-*Bt*-Cry1F Quantitative ELISA Kit (Agdia^®^, Indiana, USA). Samples from each egg mass in each treatment (100 μl) were transferred to the ELISA plates in quintuplicate to obtain precise estimates for each egg mass (average estimated egg mass size = 259 eggs). Each of the 15 plates had Cry1F standards for a calibration curve of 0 (four wells/plate), 2.5 (three wells/plate), 5 (three wells/plate), 10 (three wells/plate) and 15 (three wells/plate) ng/well. The Cry1F used as the standard was produced by Dr. Pusztai-Carey (Department of Biochemistry, Case Western Reserve University, Cleveland, Ohio) and was purchased purified and trypsinized, similar to the active form expressed by *Bt* maize. ELISA was conducted according the manufacturer’s instructions, and quantification was done by measuring absorbance at 630 nm in an iMark Microplate Absorbance Reader (Bio-Rad^®^, California, USA). Each plate was read twice. To verify the expression of Cry1F in the *Bt* maize, one gram of leaf samples (fresh weight at the V8 stage) was collected from three *Bt* and non-*Bt* maize plants, and Cry1F was estimated by ELISA as above.

### Statistical analysis

Quantification of the Cry1F protein in each sample was estimated first by averaging the two reads for each well, subtracting the absorbance for the blank (no sample controls), converting absorbance to ng Cry1F per well using the average calibration curve from all of the plates (Table B in S1 File), and correcting absorbance values for plate effects. This was converted to ng Cry1F/10 eggs using the number of eggs per well. Technical replicates were averaged, and any negative values set to 0. To reduce additional noise, positive daily estimates for the control treatment were subtracted from the corresponding values for the three exposed treatments. The detection threshold (limit of detection, LOD) was calculated using 3X the standard deviation of the blanks (*s*_*b*_), which was 0.421 ng Cry1F per well. Precision was estimated empirically from the five technical replicates for each of the 200 egg masses in all of the treatments, and was 0.570 ng Cry1F per well. This means that estimates that differed by 0.570 ng/well or more were quantitatively different. The precision was similar to the LOD. Continuous data were analyzed by ANOVA with treatment as a factor, and day of oviposition as a repeated measure using Proc GLM in SAS 9.4. Means of the significant interaction effect between the treatment and the day of oviposition were separated by Tukey´s HSD test. Binomial data were analyzed by logistic regression using the Wald Chi-Square, with treatment as a factor using Proc Logistic in SAS 9.4, and means were separated by Wald contrasts.

## Results

### Confirmation of susceptibility and resistance in *S*. *frugiperda* populations

To test if the *S*. *frugiperda* populations were susceptible or resistant as supposed, we estimated the larval survival of both populations by exposing them to feed on TC1507 *Bt* maize leaves expressing Cry1F. We verified that susceptible larvae that fed on *Bt* maize leaves expressing Cry1F had 0% survival, while susceptible larvae not exposed and resistant larvae exposed and not exposed to Cry1F had high and similar survival (Table 1). These results showed that the resistant population was resistant and the susceptible population was susceptible to Cry1F. We also found that Cry1F had no detectable detrimental effect on female reproduction and longevity (Table A, Fig A, and Fig B in S1 File).

**Table 1:** Survival (±SE) of *Spodoptera frugiperda* exposed or not to Cry1F as larvae from ten days after eclosion until pupation (*n* = 9). Resistant larvae were from Leite et al. [18] population and susceptible from SUS population described by Farias et al. [1,5].

Treatments	% larval survival
Resistant not exposed	94 ± 2.2 a
Resistant exposed	90 ± 2.8 a
Susceptible not exposed	86 ± 3.3 a
Susceptible exposed	0 ± 0.0 b

Treatment effect: *F*_3,32_ = 133.76, *P* = 3.49 x 10^−18^. Means followed by the same letter are not significantly different according to the Tukey’s HSD test.

### Cry1F detection in the eggs

The TC1507 *Bt* maize event expressed Cry1F in V8 leaves at 207.9 ± 5.86 ng Cry1F/mg fresh weight of leaf. No Cry1F was detected in the eggs of the unexposed susceptible treatment. For the 50 control egg masses measured, the estimate obtained was 0.76 ± 0.095 (SE) ng Cry1F/10 eggs [0.25 ± 0.021 (SE) ng/well]. This was consistently below the LOD and about half as large as the detections we reported in the other treatments.

Cry1F was detected in the eggs at 1.47–4.42 ng/10 eggs, when only one or both sexes of the resistant parents were exposed to Cry1F during the larval stage (Table 2). There were more observations above the LOD and above the precision level when one or both parents were exposed than unexposed, and more when both parents were exposed than when only one parent was exposed. There was a consistent detection of Cry1F in the egg masses of parents exposed to Cry1F. As 10 eggs weighed about 0.59 mg, egg concentrations were 28–83 times less than in the leaf. However, there was approximately 1500–2500 eggs laid per female during the first five days of the oviposition period, so about 212–1103 ng Cry1F were transferred to the eggs during this time. Thus, *S*. *frugiperda* was able to transfer to offspring a considerable amount of Cry1F from its host plant. As detection relied on ELISA, it was not known if intact Cry1F was detected, but in a previous study [12] on another lepidopteran species using ECL-Western Blot, it was shown that intact Cry protein was sequestered and transferred to offspring.

**Table 2:** Cry1F detected (ng/10 eggs) and percent of the technical replicates greater than the limit of detection (LOD) and greater than measurement precision in the first five egg masses of *Spodoptera frugiperda* exposed or not to Cry1F (±95% CI) as larvae from ten days after eclosion until pupation (*n* = 10 couples/treatment, 5 egg masses per couple, 5 technical replicates per egg mass).

Treatment	ng/10 eggs	Percent > LOD	Percent > Precision
Resistant ♀ and ♂ exposed	4.42 ± 0.994 a	68.7 a	44.4 a
Resistant ♀ exposed and ♂ not exposed	1.47 ± 0.192 b	50.0 b	28.3 b
Resistant ♀ not exposed and ♂ exposed	2.86 ± 0.444 b	41.3 b	24.3 b
Susceptible ♂ and ♀ not exposed	0.76 ± 0.095 c	25.5 c	15.8 c

Treatment effect: ng/10 eggs, *F*_3,36_ = 7.58, *P* = 4.69 x 10^−4^, means followed by the same letter did not differ according to the Tukey’s HSD test; Percent > LOD, *W*_*T*_ = 89.54, *d*.*f*. = 3, *P* = 2.75 x 10^−19^, means separated by Wald contrasts; Percent > precision, *W*_*T*_ = 49.71, *d*.*f*. = 3, *P* = 9.21 x 10^−11^.

Cry 1F was detected in the eggs when either the mother or father was exposed as larvae, and more Cry1F was detected in the eggs when both sexes were exposed (Table 2). These results suggest that both parents contributed independently to intergenerational transfer. The number of egg masses per day was similar among the treatments (Fig A in S1 File).

**Fig 1:**
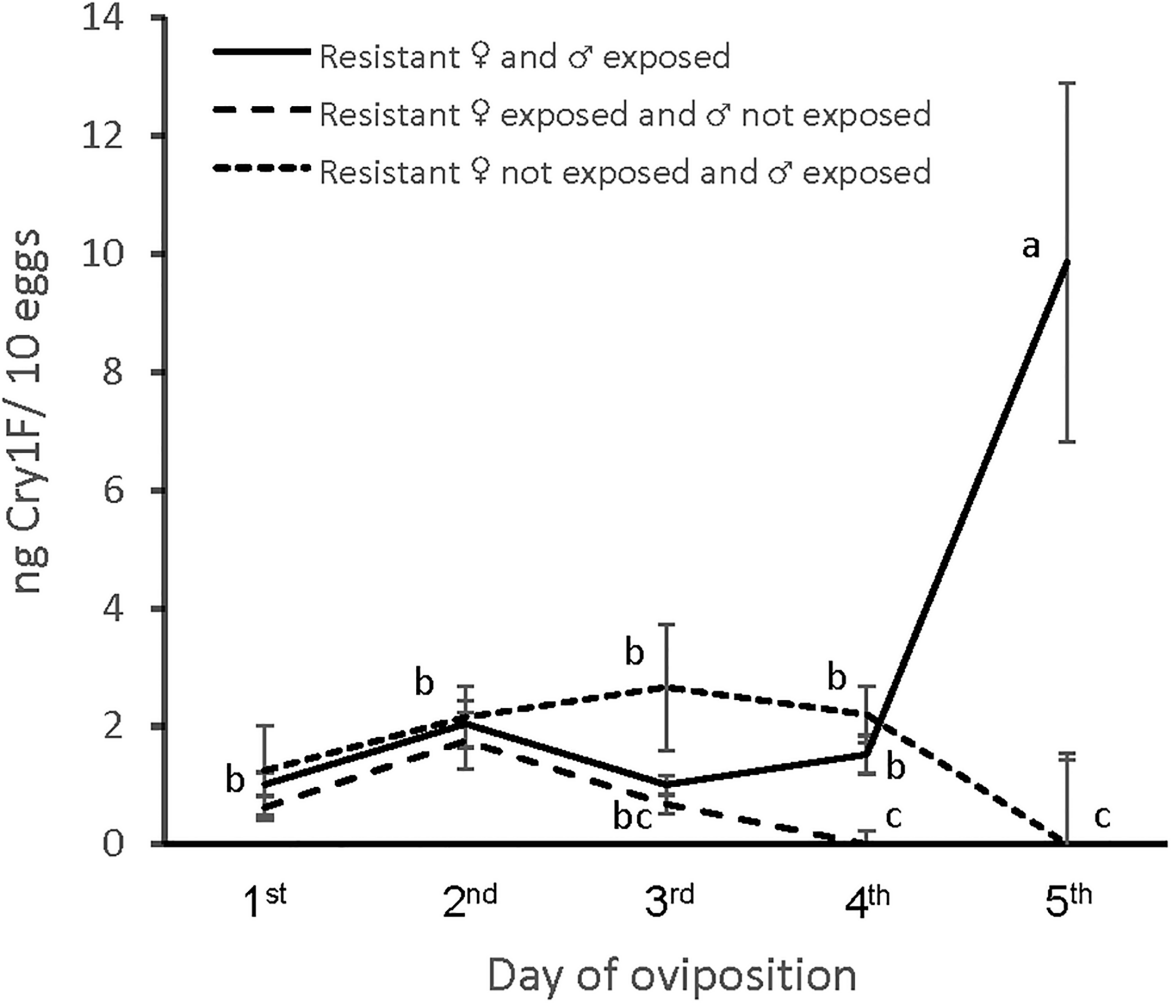
Cry1F concentration (ng/10 eggs ± 95% CI) detected in *Spodoptera frugiperda* eggs when parents were exposed or not to Cry1F as larvae from ten days after eclosion until pupation (*n* = 10 couples/treatment). Treatment x day of oviposition effect: *F*_12,144_ = 5.17, *P* = 3.57 x 10^−7^. Means for the treatment x day of oviposition interaction followed by the same letter did not differ according to the Tukey’s HSD test.

There was no significant difference in the amount of Cry1F detected in the eggs if male or female parent was the only sex exposed. Examining the detectability of Cry1F in the eggs during the first five days of the oviposition period, when both parents were exposed to Cry1F, the highest amount was detected on the fifth day of oviposition (Fig 1). When only one of the parents was exposed, Cry1F was detected only during the first three or four days of the oviposition period.

## Discussion

This study is unprecedented in demonstrating that larvae of a non-aposematic species (*S*. *frugiperda*), but resistant, can transfer a Cry protein (Cry1F) expressed in *Bt* maize leaves to its offspring eggs. Uptake and transfer of secondary plant compounds produced for plant defense is quite common in insects, particularly in aposematic species [1]. For example, larvae of the danaine butterfly *Idea leuconoe* acquire pyrrolizidine alkaloids (PA), store them throughout all stages of their life and ultimately pass them to the eggs [4]. More recently, it has been shown that Cry proteins can be taken up and transferred to offspring, and their presence are not simply transitory in the insect gut as commonly assumed [21].

Zhang et al. [10] detected Cry1Ac/Ab in unfed coccinellid neonates of the aposematic coccinellid predator *Propylaea japonica* when their parents fed on aphids reared on *Bt* cotton variety NuCOTN 33B. Gao et al. [11] detected Cry1Ab in newly eclosed hymenopteran parasitoid adults *Anagrus nilaparvatae* reared on eggs of the planthopper *Nilaparvata lugens* that fed on *Bt* rice. The parasitoid could have acquired Cry1Ab only from the eggs, indicating that *N*. *lugens* transferred the protein to its eggs. Neither of these studies was specifically designed to study intergenerational transfer, but in the first purposely designed study, Paula et al. [12] detected Cry1Ac in the offspring eggs of the aposematic lepidopteran *Chlosyne lacinia* after parental consumption of Cry1Ac, as larvae (at low concentration, LC10) or adults. Moreover, in the eggs it retained its molecular mass and toxicological activity and caused significant neonatal mortality and retarded development. In another study, Paula et al. [13] detected Cry1F in the parents and offspring eggs and unfed neonate larvae of the aposematic aphidophagous coccinellid predator *Harmonia axyridis*, in which the parents consumed, as adults, aphids (*Myzus persicae*) that fed for ≥24 h on a holidic diet containing Cry1F.

The finding that both sexes of resistant *S*. *frugiperda*, a non-aposematic generalist herbivore, are also able to transfer Cry protein may indicate that the these processes are not restricted to aposematic insect species. It was not the scope of the current work to elucidate the physiological routes that enable these processes. Indeed, the process of Cry protein uptake from the insect gut lumen into the hemolymph remains obscure. A possible route of Cry protein uptake through the insect midgut might be related to a mode of toxin tolerance introduced by Rahman et al. [22,23] and developed by Ma et al. [24], referred to as glycolipid-mediated toxin sequestration. In this mode of toxin tolerance, monomeric Cry proteins in the lumen of the midgut can bind to glycolipids, forming tetramers and aggregating in association with the gut membrane, preventing interaction with cadherin-like receptors. Protein movement from the hemolymph into the fat body by pinocytosis is common and was first demonstrated using a foreign plant peroxidase as a tracer [25], which indicated that this process is somewhat non-specific. Insect fat body stores proteins in granules throughout the larval stage before pupation as a reserve for new adult tissues [26–28].

Protein movement from the maternal adult hemolymph into the oocytes has been demonstrated at the time of yolk formation in insect eggs [25,29–34]. Many proteins in the oocyte were antigenically indistinguishable from proteins occurring in the maternal hemolymph [29,30]. In addition, foreign proteins (*e*.*g*., bovine serum albumin—BSA, fluorescein-labeled rabbit serum globulin—FSG) injected into the hemolymph before yolk formation subsequently were detected in the insect oocyte [30,34]. Many of the egg proteins originate from the parental fat body and enter the oocyte by way of the hemolymph [30] in what appears to be a non-selective process through pinocytosis [30,35], although some proteins, such as vitellogenin, are preferentially taken up by the oocyte [36].

Protein movement in males may follow similar pathways as for females, moving from the hemolymph to the fat body, where they might be mobilized into the male reproductive system and passed to females during insemination and used by females during oogenesis. It is well known that secondary plant compounds are passed to females during insemination and females incorporate these compounds into their eggs. For example, in the arctiid moths *Utetheisa ornatrix* and *Cosmosoma myrodora*, males pass a large fraction of their acquired PA to the females via seminal fluids, and females transfer these PAs to her eggs [37,38]. Less is known about proteins, but males produce a protein-rich seminal fluid [39], which is passed to females during insemination. In a grasshopper, some of these proteins were incorporated intact into eggs during vitellogenesis [40], and this may also occur in Lepidoptera [41]. In two related noctuids, proteins in seminal fluids were passed to females that incorporated them into the surface of fertilized eggs [42]. Thus, the observed paternal Cry1F transfer to eggs suggests that males may pass the protein to their mates during insemination and females may incorporate it in the eggs.

The initial increase followed by a decrease in the concentration of Cry1F in the eggs when only a single sex was exposed also was observed in the aposematic *H*. *axyridis* [13]. These results indicated that transfer was a transitory process, and limited by the amount of Cry1F uptaken by a parent. Therefore, Cry1F concentration in *S*. *frugiperda* eggs were probably related to multiple factors, such as resistance to the toxin, period that the parents were exposed as larvae, parental sex exposed, and level of Cry1F expression in the host plant. If the ability of the target pest to transfer Cry1F from the *Bt* plant to the descendants is a pleiotropic effect of *Bt* resistance, it might be expected that Cry1F transfer would vary among populations of *S*. *frugiperda* in relation to the level of resistance. Otherwise, it might be an additional selection pressure for resistance. The implications that these processes may have on insect resistance evolution and management of *Bt* crops remain unknown, but the consequences of Cry1F transfer need to be investigated. Pest resistance is one of the major undesired effects of the continuous expression of *cry* genes in *Bt* crops [43] and it is considered one of the major threats to sustainable use of *Bt* technologies.

## Supporting information

S1 File Evaluation of potential detrimental effect on female reproduction and longevity; ELISA calibration curve; Figs A and B. (DOCX)
