# Supplementary material for: Transfer of Cry1F from Bt maize to eggs of resistant Spodoptera frugiperda
Source: PLoS One. 2018 Sep 12;13(9):e0203791. doi: 10.1371/journal.pone.0203791 (PMC6135484; doi:10.1371/journal.pone.0203791)
Supplement: S1 File — (DOCX) [file pone.0203791.s001.docx]

**SUPPORTING INFORMATION**

**Transfer of Cry1F from *Bt* maize to eggs**

**of resistant *Spodoptera frugiperda***

Camila S. F. Souza, Luís C. P. Silveira, Débora P. Paula, David A. Andow, and Simone M. Mendes

**Evaluation of potential detrimental effect on female reproduction and longevity**

We evaluated potential detrimental effects of Cry1F on female reproduction by recording the duration of the pre-oviposition and oviposition periods, total number of egg masses laid per female, number of egg masses laid daily per female for each couple, and female longevity. The number of eggs per egg mass was estimated based on the average weight of 10 eggs measured independently ten times. Larval survival (angular transformation), pre-oviposition and oviposition periods, female longevity, and total number of egg masses were subjected to a one-way analysis of variance (ANOVA). Treatment means were separated using Tukey´s Honestly Significant Difference (HSD) test. The number of egg masses laid per day per female and the number of eggs per egg mass per day were analyzed with a repeated measure ANOVA with treatment as a factor, day of oviposition as the repeated measure, and mating pair as a random effect to comprise the appropriate error terms. Means within days of oviposition were compared using 95% confidence intervals. Only the treatments with resistant parents were analyzed because the susceptible population was not isogenic to the resistant population. These analyses were done with Proc GLM in SAS 9.4.

No significant detrimental effects were observed (Table A). The total number of egg masses laid per female was lower when only the resistant male parent was exposed as larvae. The peak of egg mass production for the treatments occurred between the fourth and sixth days of oviposition, descending afterwards (S1 Figure, Day of oviposition effect: *F*_14,714_ = 27.79, *P* = 2.206 x 10^-58^). There was no difference in the treatment averages (*F*_2,51_ = 2.65, *P* = 0.0807) and there was no significant variation in the temporal pattern of egg mass deposition among the treatments (Treatment x day of oviposition effect: *F*_28,714_ = 0.95, *P* = 0.5345). The number of eggs per egg mass differed among the treatments (*F*_2,27_ = 9.80, *P* = 0.0006), with fewer when only resistant males were exposed (Figure S2). There were significant differences related to day of oviposition (*F*_4,108_ = 6.76, *P* = 6.80 x 10^-5^), but no significant interaction between treatment and day of oviposition (*F*_8,108_ = 1.70, *P* = 0.1068).

**Table A**. Pre-oviposition and oviposition period duration, longevity (females) and total number of egg masses laid per female (±SE) of *Spodoptera frugiperda* exposed or not to Cry1F as larvae from ten days after eclosion until pupation (*n*=10 couples/treatment).

| **Treatments** |  | **Pre-oviposition (days)** | **Oviposition (days)** | **Longevity (days)** | **Total # egg masses/female** |
| --- | --- | --- | --- | --- | --- |
| Resistant ♀ and ♂ exposed |  | 1.7 ± 0.2 a | 6.6 ± 0.5 a | 10.1 ± 0.6 a | 10.5 ± 1.0 a |
| Resistant ♀ exposed and ♂ not exposed |  | 2.0 ± 0.2 a | 6.1 ± 0.4 a | 9.7 ± 0.5 a | 10.1 ± 0.5 a |
| Resistant ♀ not exposed and ♂ exposed |  | 1.6 ± 0.2 a | 6.2 ± 0.4 a | 9.8 ± 0.6 a | 7.5 ± 0.5 b |
| *F*_2,27_ |  | 0.94 | 0.34 | 0.14 | 5.20 |
| *P* |  | 0.4046 | 0.7118 | 0.8715 | 0.0123 |

Means followed by the same letter do not differ for the same variable according to the Tukey’s HSD test.

**ELISA calibration curve**

We conducted preliminary work using serial dilutions of Cry1F to select the concentrations that the ELISA reader would measure in the linear part of the instrument response. Each of the 15 plates had 4 blank wells, and 3 wells for each calibration concentration. The absorbance was read twice using the same ELISA reader in immediately successive measurements. For each set of the 5 replicate plates with two replicate reads for all the samples, we used Grubb’s G to identify outliers for the blanks and calibration concentrations; 4 of 480 readings were excluded, which excluded only one of the technical replicates. The variance associated with each of the calibration concentrations was estimated by ANOVA, using plate as a blocking factor. The residual variation is related to the variation within the plate for different wells and variation in measurement for the two readings of one well. This variation is reader error and variation among technical replicates.

We averaged the two readings of the plate and calculated the calibration curve using the wells from all 15 plates with an inverse variance weighted regression using SAS Proc Reg. The adjust *r*^2^ = 0.9403 (Table B).

| **Table B.** Parameter estimates, inverse variance weighted regression. | | | | | |
| --- | --- | --- | --- | --- | --- |
| **Variable** | ***df*** | **Parameter Estimate** | **Standard Error** | ***t* value** | **Pr > \|*t*\|** |
| **Intercept** | **1** | 0.04848 | 0.00184 | 26.35 | 2.216E-72 |
| **Slope** | **1** | 0.03742 | 0.00072152 | 51.86 | 2.429E-131 |

The standard deviation of the blanks was *s_b_* = 0.140 ng Cry1F/well, so the LOD = 3 *s_b_* = 0.421 ng Cry1F/well (Currie 1968, 1980, 1983; Currie & Horwitz 1994). The average egg mass size was 259 eggs/well.


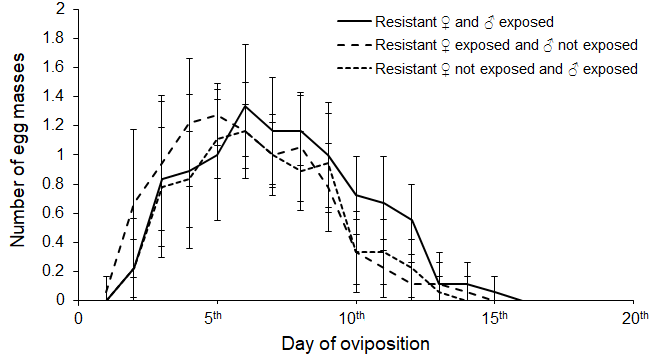


**Fig A.** Mean daily number of egg masses laid per female of *Spodoptera frugiperda* exposed or not to Cry1F (±95% CI) as larvae from ten days after eclosion until pupation (*n*=18 couples/treatment). There were no differences among means for any.


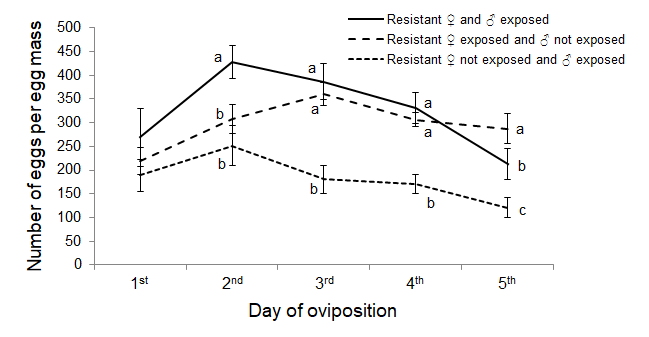


**Fig B.** Estimated number of eggs per egg mass per female of *Spodoptera frugiperda* exposed or not to Cry1F (±95% CI) as larvae from ten days after eclosion until pupation (*n*=10 couples/treatment). Means were separated for each day of oviposition by non-overlap of the 95% CIs. Means with the same letter were not different; days of oviposition without letters had no significant differences.

**References cited**

Currie LA. 1968. Limits for qualitative detection and quantitative determination. Anal. Chem. 40: 586–593.

Currie LA. 1980. Guidelines for data acquisition and data quality evaluation in environmental chemistry. Anal. Chem. 52: 2242–2249.

Currie LA. 1983. Principles of environmental analysis. Anal. Chem. 55: 2210–2218.

Currie LA, Horwitz W. 1994 IUPAC recommendations for defining and measuring detection and quantification limits. Analysis Magazine 22: M24–M26.
